# Innovative Trends in Modified Membranes: A Mini Review of Applications and Challenges in the Food Sector

**DOI:** 10.3390/membranes14100209

**Published:** 2024-09-28

**Authors:** Nicole Novelli do Nascimento, Carolina Moser Paraíso, Luiza C. A. Molina, Yuliya S. Dzyazko, Rosângela Bergamasco, Angélica Marquetotti Salcedo Vieira

**Affiliations:** 1 Postgraduate Program in Food Science, Centre of Agrarian Sciences, State University of Maringa, Maringa 87020-900, PR, Brazil; nicolenovelli002@gmail.com; 2 Department of Chemical Engineering, State University of Maringa, Maringa 87020-900, PR, Brazil; pg70630@uem.br (C.M.P.); lcm.lcam@gmail.com (L.C.A.M.); rbergamasco@uem.br (R.B.); 3 V.I. Vernadskii Institute of General and Inorganic Chemistry of the NAS of Ukraine, Acad Palladin Ave. 32/34, 03142 Kyiv, Ukraine; 4 Department of Food Engineering, State University of Maringa, Maringa 87020-900, PR, Brazil; amsvieira@uem.br

**Keywords:** membrane, membrane modification, fouling, food science, nanotechnology, compounds recovery

## Abstract

Membrane technologies play a pivotal role in various industrial sectors, including food processing. Membranes act as barriers, selectively allowing the passage of one or other types of species. The separation processes that involve them offer advantages such as continuity, energy efficiency, compactness of devices, operational simplicity, and minimal consumption of chemical reagents. The efficiency of membrane separation depends on various factors, such as morphology, composition, and process parameters. Fouling, a significant limitation in membrane processes, leads to a decline in performance over time. Anti-fouling strategies involve adjustments to process parameters or direct modifications to the membrane, aiming to enhance efficiency. Recent research has focused on mitigating fouling, particularly in the food industry, where complex organic streams pose challenges. Membrane processes address consumer demands for natural and healthy products, contributing to new formulations with antioxidant properties. These trends align with environmental concerns, emphasizing sustainable practices. Despite numerous works on membrane modification, a research gap exists, especially with regard to the application of modified membranes in the food industry. This review aims to systematize information on modified membranes, providing insights into their practical application. This comprehensive overview covers membrane modification methods, fouling mechanisms, and distinct applications in the food sector. This study highlights the potential of modified membranes for specific tasks in the food industry and encourages further research in this promising field.

## 1. Introduction

Membrane technologies have assumed crucial roles in various industrial applications, including wastewater treatment [1,2,3], food sector [4,5], pharmaceutical [6], petroleum [7], compound extraction, recovery and/or concentrating [8,9] among others that demand the separation and/or purification of an initial mixture containing both unwanted and desired components [10]. Membranes are delineated as a barrier that separates two phases and restricts the transport of one or more chemical species present in the phases, either totally or partially, based on their physical and chemical properties, when a driving force is applied across the membrane [11]. This process offers numerous advantages due to its heightened energy efficiency, selectivity, compactness, low space requirement, operational simplicity, and ease of automation [12]. The separation efficiency of a membrane is determined by its morphology and composition, interactions between feed components and the membrane surface, along with process parameters such as pressure, velocity, temperature, and pH [13]. Additionally, commercially available synthetic membranes are predominantly polymeric, primarily due to their cost-effectiveness, serviceability resulting from flexibility and elasticity, and diversity of chemical and physical attributes. On the other hand, inorganic membranes provide an extended lifespan and easier cleaning, albeit at the expense of generally involving significantly highercosts [11].

Recent research has primarily focused on exploring strategies to mitigate fouling, a critical limitation in membrane processes that leads to a gradual decline in permeate flux over time, thereby incurring higher costs due to increase of energy consumption and cleaning frequency [14,15,16,17]. The IUPAC describes fouling as “the process that leads to a decline in the performance of a membrane, brought about by the deposition of suspended or dissolved solids on the external membrane surface, within the membrane pores, or on the membrane pores themselves” [18]. The deposition and accumulation of foulants results in the decrease of selectivity and permeability, ultimately leading to reduced lifespan of the membrane structure. Mitigating fouling is crucial for ensuring sustained efficiency, as it has adverse effects on membrane performance and integrity, imposing a significant challenge as the strategies and techniques must be customized to the specific fouling type and occurrence for a successful endeavor [14]. This phenomenon is particularly noteworthy in applications within the food industry, given the organic complexity of streams in this industrial sector [19]. Anti-fouling strategies include alterations in process parameters, such as pH, temperature, and module configurations, or direct modifications to the membrane, such as the incorporation of anti-fouling compounds into the membrane bulk or surface to induce effects like electrostatic repulsion, oxidation, reduction, etc. [20].

Furthermore, with the increasing consumer demand for healthy and natural products, membrane processes have assumed a pivotal role in the development of new formulations featuring antioxidant and anti-aging properties [4]. These trends are anticipated to gain momentum in the forthcoming years across diverse sectors of the food industry and this has prompted the exploration of eco-conscious approaches in membrane processes, exemplified by innovative applications such as on-farm cheese making and fruit juice concentration utilizing solar dryers equipped with membrane pouches [21]. This surge is closely intertwined with growing environmental concerns, particularly pertaining to energy consumption and wastewater management, which loom as paramount challenges for the forthcoming decades, emphasizing the potential contribution of microbial, plant, and animal organic matter recycling to a more sustainable environment [22,23]. In addition, the food industry requires large amounts of clean, potable water and produces a considerable amount of wastewater during agri-food raw material processing [24]; thus, due to scarcity of clean water, water recirculation is crucial [23]. Integration of membrane processes, when appropriately implemented, can offer economically viable and environmentally sustainable solutions for agri-food industries, with the capacity to achieve high recovery of added-value chemicals from various streams by low energy consumption and a circular processing economy [25,26].

Given these considerations, there exists a research gap in membrane technology. First, numerous works devoted to membrane modification are known. However, many of them describe modification methods, composition, morphology, and some functional properties, for instance, permeability towards calibrating substances. The works devoted to membrane technologies in the food industry mainly refer to commercial unmodified membranes. Regarding modified membranes in the food industry, the experimental data are sparse and scattered. To increase the efficiency of separation processes, usually, the operation conditions and membrane modules are optimized. The aim of this work is the systematization of the information about the usage of modified membranes in food industry, to contribute to enablingtheir wide practical application in a near future.

The present review explores the modification of commercially available membranes for their application in the food industry, including modifiers used, main results and conclusions achieved, and current trends. An overview of membrane processes, descriptions of fouling types and mechanisms, and a discussion of methods of membrane modification are given as an introduction. Subsequently, the selected research studies are categorized based on their distinct applications, such as protein recovery/purification, oil industry, extraction of bioactive compounds, applications within the juice and beverage industry, etc. This articleprovides a comprehensive overview to guide research and project management in this field, supplying better understanding of methods employed, outcomes obtained, challenges encountered, and the overall applicability of membrane modifications.

## 2. Methodology

**Figure 1:**
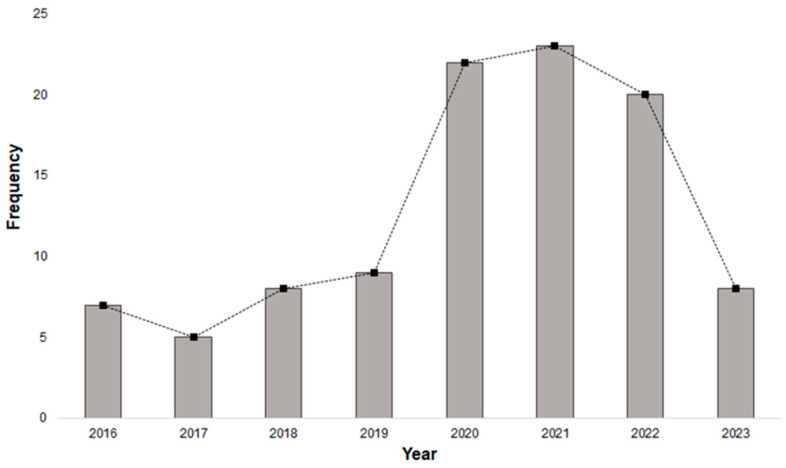
Annual publications on modified membranes with application in food areas from 2016 to 2023. Bars and dashed line reflect annual growth through 2022 and decline in 2023 are highlighted.

**Figure 2:**
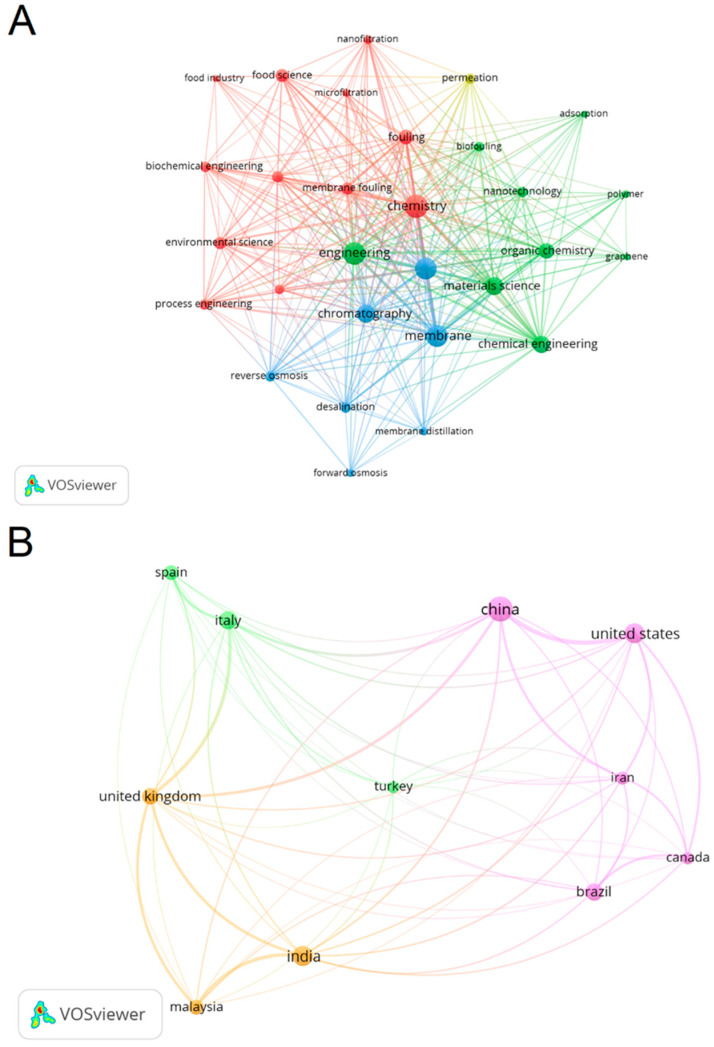
Keywords utilized in chosen articles and central countries whose authors published in the modified membrane field within the food sector between 2020 and 2024 are depicted: (**A**) keyword map indicating five points of similarity among articles; (**B**) collaboration network map among countries identified with a minimum of five citations.

This research conducted a comprehensive bibliometric review to assess the current state of research on the potential application of modified membranes in the food sector. Utilizing the Web of Science (WoS) database (https://www.webofscience.com/, accessed on 22 November 2023), a recognized excellent tool for generating citation data [27], the analysis aimed to provide insights into the scholarly landscape. The bibliometric analysis initiated with the identification of a relevant publication database. A search for the keywords “modified membrane” and “food” in documents from 2016 to 2023, categorized as “articles” and “review articles”. The research was conducted considering only English-language documents, following manual classification based on the “abstract”, totaling 102 publications. The search technique employed for pinpointing the most pertinent data, along with the analysis methods and research inquiries, is detailed in Figure 1 and Figure 2. The software tools utilized for executing and organizing the bibliometric analysis were VOSviewer (version 1.6.18) available at https://www.vosviewer.com/ (accessed on 29 January 2024). Articles prior to the years mentioned in the research methodology were used only as a theoretical basis for the literature.

## 3. Membrane Processes

In this section, the different membrane processes are presented according to their driving force, mechanism of transport, pore size, exclusion cut-off, applications, advantages, and challenges. Table 1 summarizes the characteristics of each process along with references to guide the reader to any specific knowledge required.

### 3.1. Microfiltration and Ultrafiltration

**Figure 3:**
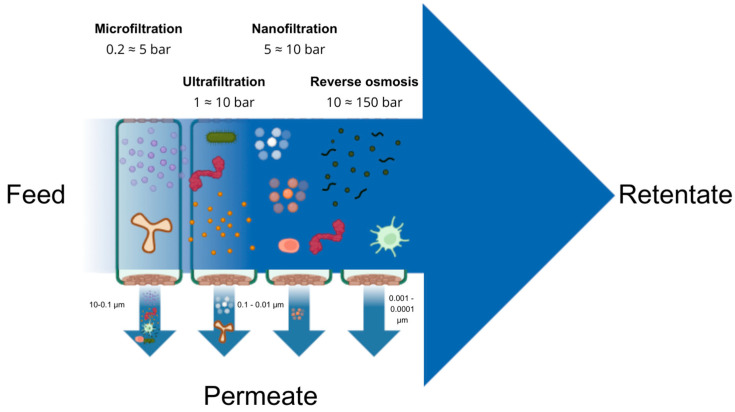
Application of pressure-driven membrane separation for removal of different species from liquids.

In microfiltration (MF) processes, where the pore sizes range between 0.1–10 μm, the primary transport mechanism is size exclusion, selectively removing suspended solids, including bacteria and basically any particles that exceed the pore size range (Figure 3). Similarly, in ultrafiltration (UF), with a pore size ranging from 0.01 to 0.1 μm, size exclusion remains the dominant mechanism and the process targets macromolecules like proteins, polysaccharides, and viruses [11]. The separation mechanism in both MF and UF processes primarily depends on the membrane’s pore size range, mainly associated with its molecular weight cut-off (MWCO), and to a lesser extent, on factors such as molecular shape, charge, and hydrophobicity [12]. The driving force for MF and UF processes is a pressure difference that induces the passage of the desired components through the membrane while retaining larger contaminants [28]. Challenges such as fouling persist, necessitating ongoing research into innovative materials and cleaning strategies [17,26,29]. Instances of this application include employing polyethersulfone (PES) microfiltration and ultrafiltration membranes that have been enhanced with nanoparticles. These membranes are utilized in the retrieval of antioxidants within the food sector, as discussed by [30].

### 3.2. Nanofiltration, Reverse Osmosis and Forward Osmosis

As the membrane pore size decreases and the process transitions to nanofiltration (NF), with pore sizes ranging from 1 to 10 nm (see Figure 3), size-exclusion steric hindrance and Donnan exclusion become the dominant mechanisms of transport and are used mainly to remove divalent ions such as calcium [5,12]. Measuring pore size in the narrow range of NF can be challenging, leading to the MWCO being less defined, as it is between porous and dense, with up to 90% rejection of molecules by this membrane.

The investigators who develop modified nanofiltration membranes were not focused on their application specifically in the food industry. As an exception, one particular work [31] considered sugar fractionation by means of a surface-modified NF membrane. The product was obtained via precipitation of polysodiyum 4 styrene sulfonate poly diallyldimethyl ammonium chloride on polysulfone UF 50 kDa membranes. These membranes have a potential for practical application in sugar industry: they are characterized by high selectivity towards sucrose over monosaccharides. An example of reverse osmotic membrane is the composite of polysulfone/polyamide hydrophilized by plasma [32]. The membrane was applied in the concentration of pomegranate juice.

All the above processes use pressure difference as the driving force for separation to occur although the amount of pressure needed varies. MF requires pressure in the 0.5 to 2 bar range, UF from 1 to 7 bar, NF from 5 to 25 bar, RO from 15 to 80 bar, and FO uses the natural osmosis pressure gradient between feed and draw solution as the main driving force [11].

In reverse osmosis (RO), membranes are dense with no detectable pores (<0.001 μm), and the separation occurs through the solution diffusion mechanism [12,33,34]. RO is used to exclude monovalent ions, such as sodium and chloride, as well as dissolved organic molecules and the smallest microorganisms; basically, almost only water can permeate this membrane [34]. Forward osmosis (FO) is emerging as a noteworthy technology;the process facilitates water passage from the feed solution to the drawing solution through osmotic pressure differences, without the need for physical or hydraulic pressure during operation [33]. The osmotic process results in water permeation from the feed to the draw solution, leading to concentration in the feed and dilution in the draw solution, which is the opposite of RO. FO offers advantages over reverse osmosis (RO) processes, including improved energy efficiency [35]. However, the mechanism of scaling formation, a type of fouling, in FO is more complicated than in the conventional RO process [36].

### 3.3. Electrodialysis

**Figure 4:**
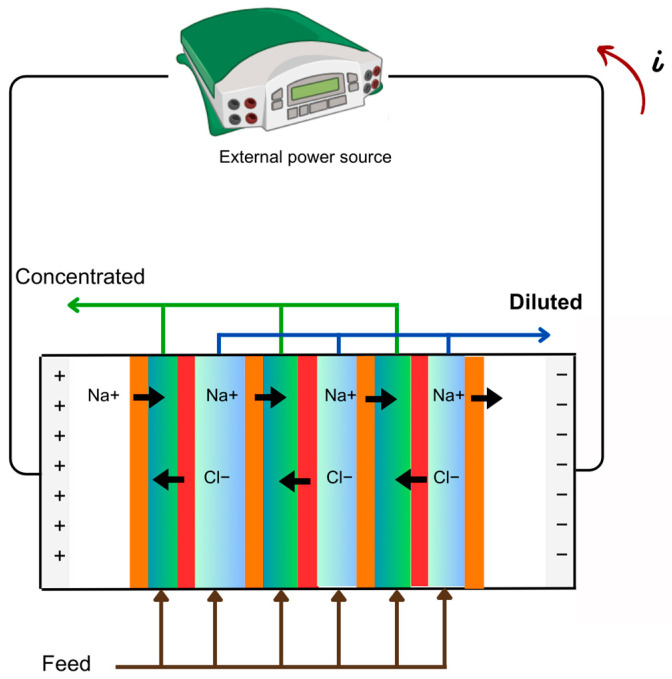
Scheme of electrodialysis: The colored bars refer to the sequential arrangement of anodes and cathodes, incorporating cation exchange membranes (orange to green—concentrate) and anion exchange membranes (red to green—concentrate). When both ions are present, the bar is represented by blue—diluted.

Electrodialysis (ED) is an alternative approach using membrane technology that harnesses electrical potential as the driving force. It employs a sequential arrangement of anodes and cathodes, incorporating cation exchange membranes (CEM) and anion exchange membranes (AEM), or, in an additional configuration, bipolar membranes composed of a cation laminate and a layer of anion exchange material [12] (Figure 4). This structural arrangement facilitates the passage of anions through AEM and cations through CEM, showcasing efficient electric charge transport through protons and water-splitting hydroxyl ions [37,38]. ED offers several advantages, including higher rates of water recovery, reduced operational costs, simplified operation, and improved membrane stability compared with RO [38,39]. Nevertheless, it comes with specific drawbacks such as membrane degradation, limitations in applicability for processes involving non-ionic compounds or molecules with low charge densities, and a complex system design influenced by the presence of competing ions in the feed solution [12,38]. The pore size falls within the same range as UF and NF membranes, aiding in the selectivity of the process, although the primary transport mechanism involves ion movement induced by electric attraction or repulsion between components and the membrane. As an example, polymer [40] and inorganic [41] membranes modified with inorganic ion-exchangers should be mentioned. These have been applied to desalination of milky whey.

### 3.4. Membrane Distillation

**Figure 5:**
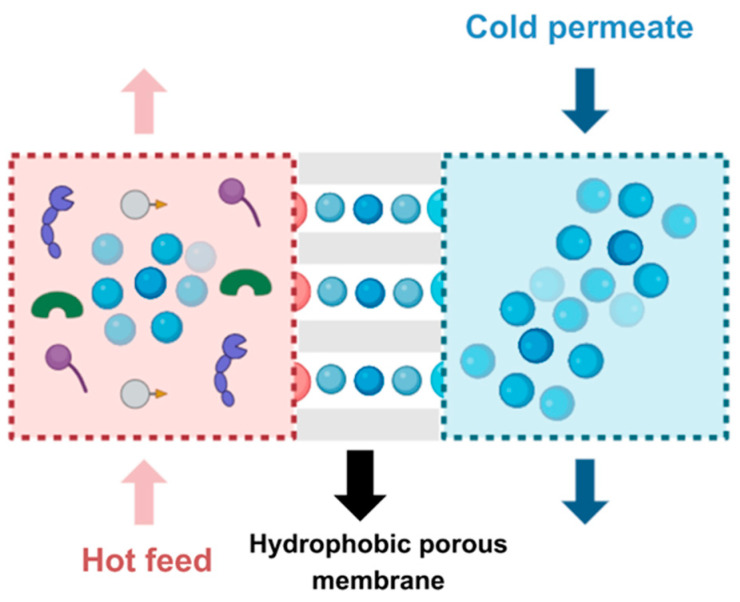
Scheme of membrane distillation: The different shapes represent solid compounds and/or microorganisms that may be present in the liquid. With the change in pressure during distillation, only vapor will pass through the hydrophobic membrane (blue circles).

Membrane distillation (MD) stands out as a thermally driven process utilizing a hydrophobic membrane. The process is operated by the difference in vapor pressure caused by the temperature variation throughout the membrane surface; the hydrophobic nature of the membrane allows only vapor to pass through, leaving liquid on the feed side and preventing it from entering the membrane pores [42] (Figure 5). The pore size range is close to that of MF and UF membranes, between 0.01–1 μm. MD presents several benefits, such as requiring less vapor space compared with traditional distillation columns, the ability to operate at lower pressures and temperatures than the feed solution boiling point, achieving a high non-volatile solvent separation factor, and being capable of concentrating aqueous solutions or producing high-purity water [42,43]. However, this process is yet to be scaled up to industrial applications, since it has acute sensibility to the feed solution [44,45]. When the permeate after cheese whey nanofiltration was treated with membrane distillation, the membrane selectivity decreased at salt concentrations of 300 g/dm^3^ and above [46]. It was found that the critical pressure, corresponding to filling the pores of the hydrophobic membrane with a liquid, was 0.6 MPa [47]. Pores wereassumed to be partially filled from both sides of membrane when the pressure was below critical value. The distance between the meniscuses of cold and hot solutions in pores decreased, enhancing the liquid transport through the membrane. Additionally, studies have underlined that the thermic gradient necessary might lead MD to incur greater operational costs compared with RO systems [44]. Composite hollowfibrous membrane, which was prepared by blending polyimide and amine-functionalized carbon nanotubes, was applied to obtainα-lecithin as emulsifier for food products [48]. However, that material did not correspond with a modified commercial membrane. In general, numerous developed composites for MD processes that have been obtained by modification of preliminarily formed membranes [49] should be tested for their application in the food industry.

## 4. Fouling

In the membrane separation process, there is generally a drop in the permeate flow at a given pressure in relation to time. The most common reasons for this problem are concentration polarization, due to the concentration of solutes on the membrane surface and is considered a reversible phenomenon [50]; and fouling, which is the deposition of solutes within the membrane pores, which may be reversible or not [51] since if the compounds are not removed from the pores, it may cause irreversible loss of membrane permeability. membrane [52]. Fouling is the biggest problem in most membrane processes. The unwanted accumulation of particles, contaminants or substances that harm the membrane surface results in a decrease in process efficiency over time and in the useful life of the membrane. The four best-known types of fouling are considering the characteristics of the fouling: particulate, inorganic, organic and biofouling.

### 4.1. Particulate Fouling

Particulate fouling involves the accumulation of suspended or colloidal particles on the membrane surface or within its pores [14,53]. Initially, the pores are partially blocked; particles accumulate near pore openings, gradually restricting the aperture. Over time, these deposited particles form layers, resulting in complete sealing and blockage of the membrane’s pores. Subsequently, increased deposition leads to the formation of a thick ‘cake layer’, compromising the membrane’s efficiency in selectively removing contaminants. This cake layer induces higher hydraulic resistance, known as cake resistance, causing concentration polarization (CP). Factors such as feed properties, operating conditions, and membrane characteristics influence particulate fouling. The mechanisms depend on particle characteristics, including size, shape, surface chemistry, and hydrodynamic conditions [53].

### 4.2. Inorganic Fouling

Inorganic fouling, also known as scaling, involves the deposition of inorganic compounds on the membrane surface or within its pores [14,54,55]. These deposits may consist of compounds with low solubility in water or solutes, present in large amounts. Inorganic fouling can occur through two mechanisms: crystallization and particulate fouling. Crystallization involves the precipitation of ions on the membrane, while particulate fouling involves the transport of particles from the solution to the membrane surface [54,55].

### 4.3. Organic Fouling

Organic fouling occurs through the adsorption of molecules like proteins, polysaccharides, and humic substances onto the membrane surface, leading to pore blockage [14,15,17]. The adsorption process is influenced by membrane properties (hydrophobicity, charge, roughness) and the organic molecules’ properties (size, shape, charge density). Foulants can induce surface chemistry changes and conformational alterations in organic molecules, enhancing their adsorption and aggregation [15].

### 4.4. Biofouling

Biofouling results from the attachment and growth of microorganisms, such as bacteria, fungi, and algae, on the membrane surface or within its pores. It is fueled by organic matter in the feed stream creating an environment conducive to microbial growth [15,56,57]. Biofilms formed by microorganisms can trap additional particles, diminishing membrane performance and increasing pressure drop. Extracellular polymeric substances (EPSs), crucial in membrane bioreactor (MBR) fouling behavior, contribute to adhesion through electrostatic interactions, hydrogen bonding, and van der Waals forces [54]. Controlling microbial activity, optimizing operational parameters, employing pretreatment methods, and implementing membrane cleaning protocols are strategies to mitigate fouling caused by EPSs [57].

### 4.5. Antifouling Strategies

Various strategies have been developed to prevent fouling and biofouling. The main approaches are to avoid or minimize concentration polarization [52]. For this purpose, micro- or ultrafiltration is carried out at low pressure, and electrodialysis is performed at low current. This approach is limited by the low efficiency of separation processes. Other methods to minimize fouling for baromembrane separarion include the manipulation of hydrodynamic conditions, particularly the use of spacers, backwashing, and backpulsing, as well as gas purging [58]. Special attention is paid to cleaning membranes, particularly to non-conventional methods, such as ultrasound activation and pulsed electric fields. Scaling can be prevented using pretreatment methods and by adding antiscalant to the liquid being purified (this approach is suitable only for water and wastewater treatment) [59]. Special pretreatment or the addition of biocidal compounds are necessary to protect membranes against biofouling [60].

Regarding electrodialysis, such approaches as turbulizing the feeding flow, pulsed electric field operational conditions, cleaning membranes and pretreatment of feeding solutions are well known [61]. For membrane distillation processes, operational conditions and composition of feeding solution have been focuses of attention [62].

A current field of study is the modification of membranes to change their permeability capacity, that is, allowing the membrane to become more hydrophilic or hydrophobic depending on the components and/or charges present in it and offering a greater possibility of different solutions being filtered or concentrated. Furthermore, the membrane can be made from more than one polymer, or another component can be used to modify it in terms of improving selectivity, flow, fouling reduction, and in economic terms. This modification can also be carried outusing solutions with components that are desired to change the membrane through the filtration process.

## 5. Membrane Modifications

Membrane separation processes have emerged as focal points in industrial applications, serving diverse purposes such as water treatment, compound recovery, and protein concentration, among others [10]. The separation performance of a membrane is affected not only by its characteristics but also by its morphology and composition, as well as process parameters such as pressure, velocity, and interactions between feed components and membrane surfaces [13]. Ongoing research in membrane processes aims to address fouling, a significant limitation in food applications involving concentrated feeds containing diverse compounds [19]. Emerging trends include in-line characterization of membrane fouling, advanced techniques like HPLC-coupled mass spectrometry and molecular simulations. Environmental concerns, particularly energy and water consumption and wastewater release, are growing, affecting major processes, including membrane processes in industries like dairy and beer. Eco-conception of membrane processes is gaining importance, with applications such as on-farm cheese making and fruit juice concentration using solar dryers with membrane pouches. As consumer demand for healthy and natural products rises, membrane processes play a crucial role in developing new formulations with antioxidant and anti-aging properties. These trends are expected to intensify in the coming years across various food industries [4].

The field of membrane modification has garnered significant attention, driven by the potential to enhance membrane selectivity, flux, and economic efficiency [62]. The alteration of membranes, achieved using multiple polymers or other components, is proving instrumental in achieving these improvements. Moreover, surface and volume modifications can be implemented by passing solutions containing desired components through the filtration process [63]. Various surface modification techniques are essential for enhancing material properties across a range of applications. These methods can be divided into physical and chemical categories [64]. Physical methods, such as coating or blending, change surface characteristics by applying thin layers or integrating different materials. Coating adds a material layer to improve features, introducing specific properties such as hydrophobicity or hydrophilicity. Blending, which is performed during material processing, can enhance mechanical properties, thermal stability, or processability [65]. Chemical methods, including polymer functionalization, plasma treatment, and graft polymerization, involve altering the material’s surface chemistry. Polymer functionalization adds specific chemical groups to tailor surface properties such as reactivity and biocompatibility [66]. Plasma treatment uses ionized gases to change surface chemistry and structure, improving characteristics like wettability and roughness [67]. Graft polymerization attaches polymer chains to a substrate to enhance properties like hydrophilicity and mechanical strength [68]. Each method offers distinct benefits and can be chosen based on the required surface properties and application needs.

### Modifying Agents

One of the most intriguing aspects of membrane modification is the ability to alter permeability, allowing membranes to become more hydrophilic or hydrophobic based on their components and/or charges. Sulfonation, involving the insertion of sulfonic groups (-SO_3_H) into the polymeric matrix, aims to increase hydrophilic characteristics, thereby reducing fouling with organic compounds [69,70]. Researchers [29] engineered a polyethersulfone (PES) ultrafiltration membrane incorporating a melamine-modified zirconium-based metal-organic framework (MOF), known as UiO-66-NH_2_. The membranes underwent testing in both oily wastewater and powdered milk solutions, showcasing robust antifouling properties. The coating method involving the repetitive application of thin layers provided an effective anti-fouling solution. Deposition mechanisms may include adhesion or adsorption through multiple interactions between functional groups, interpenetration of functional materials into the base polymer, or macroscopic entanglement of functional groups in the membrane pores. Coating methods preserve a thin selective layer on the membrane surface [71].

Graphene, a two-dimensional atomic carbon structure, has been a focal point of research due to its ability to significantly enhance the performance of many polymers, such as membrane surfaces [71,72]. The deposition of graphene oxide on membrane surfaces modifies their characteristics, reducing porosity and imparting features like increased selectivity and resistance to fouling [73]. Addressing fouling issues in membrane separation processes is crucial for sustained efficiency. Anti-fouling mechanisms involve changes in pH, temperature, and the addition of anti-fouling compounds to the membrane surface, inducing effects like electrostatic repulsion, oxidation, or reduction [20]. Alternative nanoparticles, including metal oxides like TiO_2_ and SiO_2_, can be employed to enhance the hydrophilicity of membrane surfaces. This improvement in anti-fouling performance is applicable not only in the food industry but also in water treatment [74].

A composite of hydrated zirconium dioxide–graphene oxide was applied in the modification of polyacrylonitrile-based ultrafiltration membranes [75]. The synthesis method included deposition of the composite inside the active layer of the membrane. Here, carbon nanomaterial and inorganic ion-exchanger performed the functions of hydrophilizing agents. Additionally, zirconium dioxide was a binder preventing leakage of graphene oxide from the membranes under the influence of applied pressure. The embedded modifier enhanced the separation ability of the membranes: the rejection of bovine serum albumin (69 kDa) and ovalbumin (40 kDa) reached 95–98%. Composite membranes were polluted according to the mechanism of the cake formation on the outer membrane surface. Thus, the membranes were easily cleanable by hydrodynamic pulsation or reverse permeate flow. At the same time, the pristine membrane showed selectivity of 10–22%. In addition to cake formation, the mechanism of fouling can also involve pore constriction; in such cases, chemical reagents are needed to clean membranes. As an alternative to graphene oxide, carbon nanodots were proposed as additions to hydrated zirconium dioxide [76]. Their advantage over graphene oxide is a simple synthesis procedure, which is realized under autoclave conditions and requires no aggressive reagents.

European Nanotechnology Gateway defines nanofoods as those predominantly utilizing nanotechnology techniques involving nanoparticles in the harvesting, processing, and preservation of food [77]. The incorporation of nanotechnology in the food industry holds the potential for developing innovative products [78]. Additionally, nanoparticles contribute to enhancing the physical–chemical qualities of food by reducing microbial load, affecting cell membranes, generating reactive oxygen species, and offering physical, chemical, and mechanical resistance. These nanomaterials effectively isolate and concentrate contaminants in food matrices [79,80]. The increasing demand for healthy foods underscores the necessity to modernize food processing technologies. Nanotechnology strives to enhance the biological effectiveness and physicochemical stability of bioactive compounds during food processing, thereby aiding in the creation of functional and novel food products with heightened health benefits [81].

## 6. Utilizing Modified Membranes in Food Industry

Membranes used in the food industry must satisfy a number of requirements. Let us consider them using the example of ultrafiltration membranes. First of all, a key property is the necessary high selectivity of membranes towards components. For instance, in the case of ultrafiltration of milky whey, a function of the membrane is to retain proteins. At the same time, the membrane is permeable towards such ballast components as salts and lactose. Another requirement is high permeate flux, which is impossible without the membrane’s stability against fouling and biofouling. One of the ways to solve this problem is to use modified membranes. In many cases, it is not recommended to utilize materials modified with enzymes for the treatment processing of food feedstock, since they cause degradation of target products. Nevertheless, some examples of the usage of enzyme-containing membranes are given in the literature [82], including limiting the hydrolysis of proteins, hydrolysis and synthesis of oils and fats, and polymerization of low-molecular-weight sugars. However, modified membranes of this type can be successfully used for the treatment of wastewater produced by the food industry. The same application field has been reported for membranes modified with photocatalytic agents [83].

Finally, a very important requirement for modified membranes is their chemical stability against acidic and alkaline media as well as in disinfecting solutions, which are used for rinsing membrane systems [84]. Here, we further consider the application of modified membranes in different branches of the food industry.

### 6.1. Oil Industry

In recent times, membrane separation processes have found extensive applications in various food industries, as can be seen in this review. Since the 1980s, research has been ongoing in utilizing membranes in vegetable oil technology, with continuous studies in progress [85]. Membranes are employed for oil removal, requiring materials with hydrophilic groups such as oxygenated and nitrogenated compounds, resistant to organic solvents. As organic solvents, typically highly nonpolar, are used, membrane surface interaction or degradation must be mitigated [86].

Degumming via membrane processes can be conducted with miscella or crude oil without solvent addition. However, stability issues with miscella and low permeate flux with crude oil hinder industrial-scale application [87]. Studies on sunflower and soybean oils without solvent addition, utilizing polymeric membranes, demonstrated 77% and 73.5% phospholipid retention, respectively [88]. Enhanced miscella stability using PVDF, PES, and PS ultrafiltration membranes was reported [87]. However, fouling in the oil area continues to be a challenge even with advances in modifications. This is because the surface modification approach must introduce preferred functional groups or nanoparticles on the membrane surface, imparting antifouling properties and minimizing potential risks [85]. A polycarbonate membrane enhanced by incorporating halloysite nanotubes and graphene oxide nanosheets for the removal of olive oil from an aqueous solutionwas reported [89]. Apart from enhancing flow and offering antifouling properties, the modified membranes achieved a remarkable 100% efficiency in rejecting olive oil.

Hydrophilizationof polyvinylidene fluoride (PVDF) membranes was considered for application to a saline feed solution containing mineral oil [89,90,91]. This modification involved plasma-induced polyethylene glycol (PEG) grafting and the subsequent deposition of TiO_2_ particles onto the membrane surface. The results indicated a notable decrease in oil accumulation on the surface and inside the pores of hydrophilized membranes. While initially designed for mineral oil, this approach may present an alternative for handling oily substances in the food sector. Similarly, other researchers [92] developed a membrane using a blend of cellulose acetate and zwitterionic nanoparticles for the purpose of treating water contaminated with oily waste commonly found in various industries, including the food industry. The membrane not only demonstrated a remarkable oil recovery efficiency ranging between 95–99%, but also exhibited enhanced hydrophilicity, resulting in a water flow rate of 583.64 ± 25.12 L m^−2^ h^−1^, considered suitable for the specific conditions of their study. Moreover, a reduction of almost 9% of irreversible fouling caused by oily water treatment was reported.

**Figure 6:**
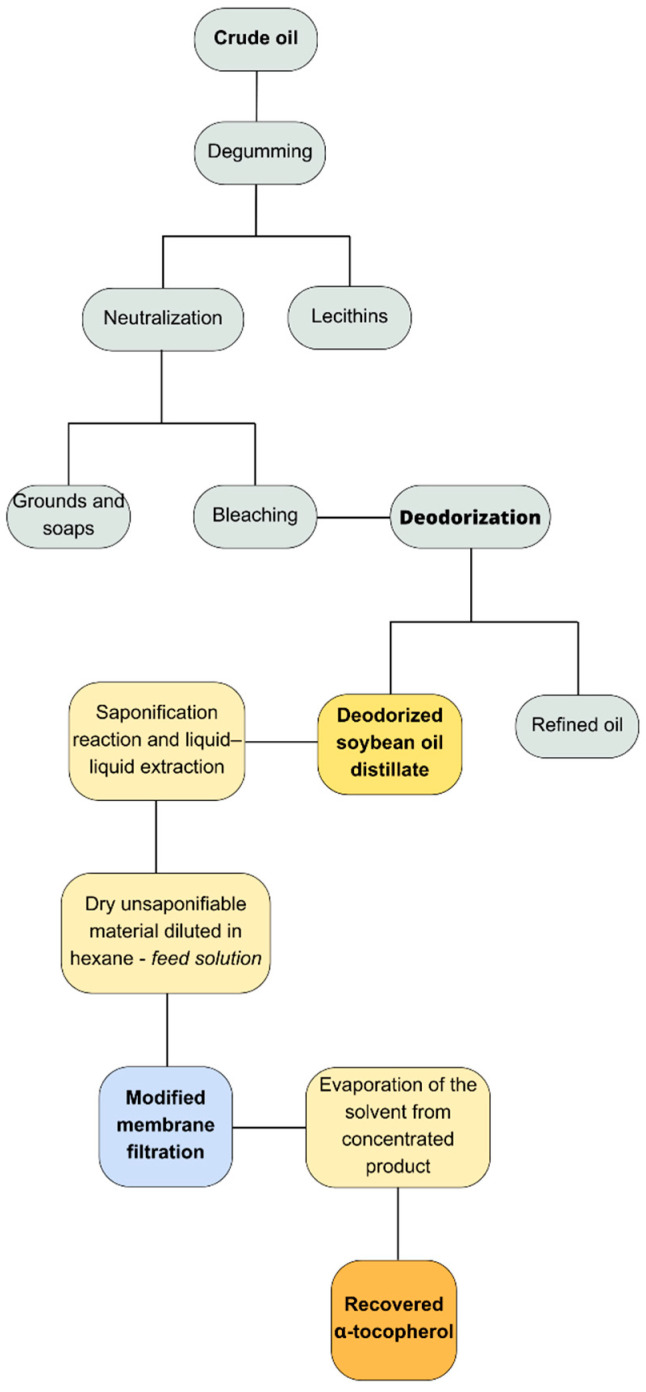
Tocopherol recovery in oil industry using modified membrane by [95].

Membrane technology has beenexplored for recovery, preservation, and concentration of tocopherols, antioxidantspresent in vegetable oils. Studies indicate permeation of tocopherols through membranes, with varying retention percentages based on oil type [92,93,94]. In [95], the use of soybean oil deodorization distillate as a tocopherol source enabled the recovery of α-tocopherol with enhanced biological activity, as in the example in Figure 6. This was achieved through a surface-modified membrane incorporating sulfonic groups, polyethyleneimine, and graphene oxide nanoparticles functionalized with tannic acid. The recovery process yielded 82.00% of α-tocopherol in the concentrate, with a flow recovery rate of 64.39% and operational capacity for two cycles. These findings underscore the effectiveness of the surface-modified membrane as an alternative to conventional methods, offering energy cost savings as well. With ongoing research, the ideal membrane for industrial-scale performance becomes a pivotal focus. Moreover, nanoparticles like graphene oxide represent a versatile and transformative technology applicable across various scientific disciplines [96].

Another application within the oil industry involves the use of biocatalysts. For instance, the immobilization of lipases on polypropylene hollow fiber microfiltration membranes to hydrolyze olive oil has been demonstrated [97]. Immobilizing lipases in the membrane increases the efficiency of the catalytic reaction, as it can increase enzymatic activity and extend its lifespan.

### 6.2. Proteins

Membrane separation is widely used for obtaining or treatment of concentrates of proteins of plant or animal origin [98,99]. Membrane modifications must meet criteria that do not compromise protein structure while ensuring mechanical stability, solvent resistance, and allowing the diffusion of other substances in cases of enzyme immobilization [100]. Hydrophilic membranes exhibit a high affinity for water molecules, reducing fouling through solubility with proteins [101]. On the other hand, some studies have suggested that hydrophobization may reduce membrane leaching and improve enzymatic activity [102].

The continual exploration of innovative membrane modification strategies holds significant potential in overcoming fouling challenges and advancing industrial processes. As an example [103], it was demonstrated that macroporous membranes, specifically those prepared with polymethylacrylate, had the capability to clarify juice and fractionate proteins into smaller fractions. That study showcased a recovery potential of 30–91% for vegetable albumin extracted from Moringa oleifera seeds using these membranes. However, to enhance flow conditions, the authors recommended the incorporation of hydrophilizing agents such as hydrophilic polymers, inorganic ion exchangers, and graphene oxide. This type of potential modification holds appeal for the food industry, as it avoids the destruction of organic substances.

Other researchers [104] enhanced microporous polyvinyl chloride membranes by introducing alumoxaneparahydroxybenzoate through the phase inversion technique. The modified membrane demonstrated exceptional hydrophilicity and enhanced selectivity when tested with bovine serum albumin solution. Subsequently, they assessed its performance in terms of whey protein reuse and concentration, revealing increased resistance to fouling and improved permeability. To obtain protein concentrate from cheese whey, polymer microfiltration membranes modified with a composite of hydrated zirconium dioxide and carbon nanodots were used [76]. The composite materials obtained in this manner demonstrated high selectivity towards the proteins in milky whey. In an alternative approach, hydrated zirconium dioxide and basic bismuth nitrate were employed to modify macroporous ceramics [42,105]. The resulting modified membrane was utilized in the desalination of whey, achieving an efficiency of 75% within 30 min, demonstrating superior performance compared with conventional methods. Addressing solutions with ionic charges, researchers [40] investigated the use of polymeric membranes enhanced with zirconium hydrophosphate nanoparticles and hydrated zirconium dioxide for ion exchange in the desalination of whey. Through electrodialysis, the membraneachieved a separation efficiency of 97% for cations such as K^+^, Na^+^, Ca^2+^, Mg^2+^ and 11–22% for anions like HPO_4_^2−^ and H_2_PO_4_^−^, underscoring the effectiveness of the method in achieving ion separation.

### 6.3. Bioactive Compounds

Extraction serves as the crucial initial phase in retrieving bioactive compounds from plant matrices [106]. In the case of citrus, various contemporary extraction techniques have been employed, including organic solvent extraction [107], alkaline compounds [108], and adsorption on macroporous resins [109], among others. However, the use of these methods may lead to compound degradation due to high temperatures and prolonged extraction times, potentially causing toxicity concerns related to the solvents utilized [110]. Consequently, the growing interest in recovering bioactive compounds from plant sources, especially fruit processing by-products, has spurred researchers to develop new extraction methods that demonstrate higher efficiency compared with traditional processes [111].

Ginseng, renowned for its medicinal properties, is extensively incorporated into diverse beverages. However, the conventional concentration process involves substantial energy consumption and results in the loss of its active compounds. Hence, [112] proposed an alternative solution using a modified membrane. The hollow fiber (HF) poly(vinylidene fluoride) (PVDF)/polysulfone (PSF) membranes exhibited an impressive 99.96% rejection rate for a ginseng extract solution, facilitating extract concentration through a contact membrane distillation process. Moreover, the membranes were enhanced with improved hydrophobic properties and heightened mechanical resistance. Further research [113] developed a microfiltration membrane composition comprising ceramic powder (60%), dimethylsulfoxide (DMSO) (33.6%), polyethersulfone (PESf) (6%), and arlacel (0.4%). This membrane was employed for the recovery and purification of bioactive compounds from hibiscus calyx extract (*Hibiscus Sabdariffa* L.). The findings indicated that under the evaluated conditions, it was possible to reduce the solid content while maintaining the antioxidant activity of the extract.

Citrus processing generates substantial amounts of residues and by-products rich in bioactive compounds. While some by-products are currently valued, the majority are still discarded, posing environmental challenges [114]. Researchers are focusing on these waste sources to obtain high-value products like antioxidants, aiding in food preservation and replacing synthetic additives harmful to health [115]. An illustrative instance involved the utilization for recovery and concentration of β-carotene from industrial carrot peel residues, offering a potential alternative to synthetic antioxidants [116]. This process involved layer-by-layer modification of the membrane with sulfonic groups, polyethylene glycol, and graphene oxide functionalized with tannic acid. The outcome yielded a purification of approximately 70% of β-carotene, exhibiting significant potential antioxidant activity as indicated by FRAP (131.30 µmol TE/g sample) and Folin–Ciocalteu (129.84 mg/L) assays.

### 6.4. Pectin

Pectin represents one of the main structural polysaccharides in the cell walls of dicotyledonous and some monocotyledonous plants. This polysaccharide has several properties that make it useful in the food industry, performing functions as a gelling, thickening, and stabilizing agent [117,118]. Currently, commercial pectin is mainly extracted from citrus fruits, such as orange and lemon, or from residues such as apple pomace and passion fruit albedo [119]. Due to growing industrial demand, there is a continuous need to improve pectin extraction and purification processes [118,119]. A studyinvestigated the application of a hydrophilic hybrid coating on the surface of a polyvinylidene fluoride ultrafiltration membrane [120]. This coating, composed of polydopamine (pDA) and (3-Aminopropy) triethoxysilane, incorporated antiscaling properties of pectin, forming a layer that presented hydrophilic groups (aminogroups, Si-OH, Si-O-Si) on the surface of the modified membrane. Filtration experiments revealed a significant increase in flux recovery rate and reversible fouling rate compared with the unmodified membrane, recording increments of 48.07% and 44.46%, respectively.

The application of kaolin membranes functionalized with graphene oxide (GO)/ethylenediamine (EDA) was evaluated [121] for the concentration of pectin from orange peel extract. To modify the hollow fiber, a simple vacuum-assisted deposition method was used in which the fiber was completely immersed in the GO/EDA dispersion under a vacuum of 50 mmHg for 10 min. After this time, the hollow fiber was removed from the GO/EDA dispersion and the vacuum was kept on for 1 min to improve GO/EDA adhesion on the fiber. A GO/EDA layer with a thickness of 2.86 ± 0.24 μm was formed. The membrane coated with GO/EDA showed greater selectivity to pectin than the pristine hollow fiber. The GO/EDA-coated hollow fiber concentrated the galacturonic acid, phenolic, and methoxyl contents at 19.5, 17.4, and 29.2%, respectively.

### 6.5. Sugar

A study was conducted wherein a ceramic membrane was enhanced by incorporating dopamine into the inner film coating [122]. This modification was aimed at rendering the membrane resistant to high temperatures, making it suitable for applications in the sugar industry. The modified ceramic membrane underwent rigorous testing, including in vitro cytotoxicity assessments, affirming its compliance with food safety standards. Additionally, the membrane was evaluated in the filtration of redissolved brown sugar syrup, demonstrating its capability to clarify sugar juices while exhibiting antiscaling properties.

The relationship between modified membranes and their impact on food-related processes was explored [123]. An isoporous membrane was derived utilizing block copolymers (BCPs), specifically poly(N-isopropyl acrylamide-co-acrylamidophenylboronic acid) P(NIPAM-co-APBA), along with double sugar dissolved in the solution. The incorporation of sugar was found to contribute to stabilizing the conformational structure of the proteins, consequently mitigating their denaturation during membrane separation processes. Moreover, the modified membrane exhibited accelerated separation of protein mixtures and demonstrated a notable reduction in fouling.

An additional instance relates to the purification of xylitol. Nanofiltration membranes using PES and SiO_2_ nanoparticles were engineered by [124], enhancing hydrophilicity and achieving a recovery rate of approximately 80% for xylitol in synthetic mixtures during rejection tests. Nevertheless, separating small sugar molecules, including saccharides, poses challenges in the food industry and biorefineries due to their complex hydrolyzate compositions, resulting in membrane fouling and reduced selectivity [125,126]. Therefore, leveraging nanoparticles with antifouling properties is emerging as an optimization strategy to address factors influencing the efficiency of such separations.

### 6.6. Fruit Juices

The presence of diverse compounds in fruit and vegetable juices poses a risk of fouling during membrane filtration, leading to significant reductions in permeate flux and overall membrane productivity. Investigating novel anti-fouling materials for membranes and optimizing integrated membrane systems are crucial research areas for expanding the application of membrane operations in juice processing [127]. Moreover, preserving the quality and nutritional content of fruit juices, along with enhancing food safety, can be achieved during the concentration and clarification processes. Conventional methods, without additional modifications, often employ membrane processes like microfiltration, ultrafiltration, and reverse osmosis, as well as osmotic distillation and membrane distillation [128,129].

Modification of FO membranes in use in the fruit juice industry can enhance hydrophilicity and solute rejection while ensuring compatibility with the selective layer and maintaining chemical stability and mechanical resistance. While quality attributes such as bioactivity, freshness, aroma, and color of fruit juices should be preserved, it is important to consider stability with regard to enzymes. Use of an FO membrane to separate extracellular enzymes from pathogenic bacteria or fungi remains a significant challenge. Additionally, achieving high concentration often requires prolonged operational times due to the low flow of FO, which can potentially promote microorganism growth and juice deterioration [130].

In prior studies, it was proven that modified poly(ether ether ketone) hollow fiber membranes successfully clarified kiwi juice. This modification induced depectinization, resulting in nearly 99% clarity and complete removal of suspended solids without significant alterations to other physical–chemical characteristics of the original juice [131]. Similarly, modified poly(ether ether ketone) and polysulfone hollow fiber membranes were employed to clarify pomegranate juice [132]. This method preserved the content of phenolic compounds, operating under improved flow conditions with reduced fouling.

Table 2 presents several studies employing modified membranes. There have been improvements in the properties of membranes, such as flow and antifouling, which have contributed to the potential reduction in the time of the filtration process.

### 6.7. Biotechnology

An illustration of this process can be found in a study where electrospinning technology was employed to fabricate a PAN nanofiber membrane [137]. Subsequent steps involved PAN hydrolysis and chitosan grafting, resulting in the creation of P-CS membranes. Qualitative analysis demonstrated antibacterial activity in all P-CS-GTMAC nanofiber membranes, particularly against *E. coli*, demonstrating a reduction of 76.7 ± 1.2% in initial bacterial activity. Additionally, the synthesized P-CS-GTMAC membranes possessed characteristics such as straightforward synthesis, cost-effectiveness, long-term stability, water stability, lack of decomposition into toxic products, and ease of regeneration.

Another aspect involves the application of biotechnology in food science. Researchers explored the elimination of ochratoxin from wine using a diatomite ceramic membrane modified with MgO nanoparticles. The removal efficiency ranged between 92–96% [138]. Despite the potential impact of this modification on the physical structure and composition of the wine’s macromolecules, it did not notably affect the flavor. This represents a novel strategy for addressing ochratoxin and other potentially harmful substances in beverages, contributing to food safety. In contrast, another group [139] implemented a surface modification on the polyvinylidene difluoride membrane by immobilizing polydopamine and nisin microspheres. This modified membrane was employed to eliminate and deactivate *Alicyclo bacillus acidoterrestris*, a microorganism responsible for significant deterioration in the quality of apple juice and challenging to treat effectively through conventional sterilization methods in food processing. This membrane achieved success in microorganism inactivation, highlighting the membrane’s antibacterial capacity and reducing the bacterial concentration by 65 times. The study underscored the significant potential of modified membranes in deactivating diverse microorganisms in liquid food processing, contributing to future perspectives on the antimicrobial activity of beverages [140].

### 6.8. Treatment Water in the Food Sector

Another noteworthy concern is the liquid waste produced by the food industry. In 2017, the European food industry alone produced 5m^3^ of wastewater daily [141]. Certain sectors within the food industry, including fish, edible oils, grain minerals, tea, and coffee, have successfully adopted ultrafiltration membranes for wastewater treatment, presenting a promising approach in support of the circular economy. Nevertheless, challenges such as registration issues and substantial initial costs persist. Consequently, a potential solution involves modifying the membranes employed in water treatments to address these issues [142].

In addition to environmental concerns, water treatment is linked to human health, as it can lead to allergies and other adverse effects like dyes [143,144]. To address this, for the removal of anionic food dyes, researchers [145] created a polyether sulfone membrane modified with sulfuric acid, polyethyleneimine, and predominantly, graphene oxide nanoparticles functionalized with tannic acid. This membrane demonstrated minimal fouling and could be effectively reused across five operating cycles, achieving a removal rate of over 80% for the tested dyes. In other research [146] microfiltration membranes using PES were developed and enhanced through the layer-by-layer self-assembly method using solutions of sulfuric acid, titanium dioxide (TiO_2_), and graphene oxide (GO). This modification demonstrated outstanding efficacy in eliminating Bordeaux red dye (approximately 93%), twilight yellow (approximately 70%), and safranin orange (100%).

Another instance involved the utilization of a PVDF membrane prepared with montmorillonite, β-cyclodextrin, tannic acid, and sodium alginate [147]. Although the primary focus was on water treatment concerning oil-water emulsions and organic dyes, the exceptional results obtained—nearly 100% removal of contaminants—suggest potential applications in the food sector.

## 7. Conclusions and Future Considerations

Modification of preliminarily formed membranes, particularly commercial materials, allows one to enhance their separation ability on the one hand and provide antifouling resistance on the other hand. This approach can sometimes be useful for customers, since it provides a possibility to obtain membranes with the necessary functional properties to solve urgent practical tasks. The membranes can be used not only for the treatment of waste water produced by the food industry, but also for the treatment of food feedstock.

Despite many works in the field of modified membranes, many of them are not focused on their application to meet the specific needs of food industry. However, many of the developed materials have a great potential for employment in this field, since many of the modifiers will not destroy the target products during modification of ultra- and microfiltration membranes, particularly in MBR processes. In the latter case, a modifier providing degradation of organic substances, for instance, a photocatalyst, can be employed. After modification, these materials can even acquire particular nanofiltration properties. These properties can be realized even under low pressure (up to 4–6 bar). The main task in this field is to provide the possibility of multiple usage of these membranes, requiringchemical and mechanical stability of the modifier during the regeneration of the membrane system. Moreover, the leakage of modifier from membranes must be excluded. However, to complete an urgent one-time task, the modified membranes may be disposable.

Modified NF, RO, FO, and MD membranes for food industry application can be considered as a prospective field of investigation. Known materials of this type should be tested to ascertain the possibility of their employment for this purpose. Modifying membranes for emulsification processes looks like an especially interesting and important task from the practical point of view. In this case, it is necessary to obtain homogeneous pores with enhanced hydrophobicity. Since many of thedeveloped modified membranes possess the necessary functional properties, their potential for food industry application should be noted. At the same time, new modifiers like metal–organic frameworks should be considered as potential substances for preparation of membranes for this purpose.

## Figures and Tables

**Table 1:** Membrane processes for the food industry. ΔP^1^: pressure difference; ΔE^2^: electrical potential; ΔP/ΔT^3^: difference in vapor pressure caused bytemperature variation; Da^4^: Dalton.

Process	Driving Force/ Reason of Retention	Pore Size Range (μm)	MWCO (Da^4^)	Advantages/Disadvantages	Some Applications in the Food Industry	References
Microfiltration	ΔP^1^/Size exclusion	0.1–10	>500,000	Low energy and pressure, easy operation and scale up, relatively low cost/Limited applications due to wide pore size, sensitive to oxidants and prone to high fouling.	Low-temperature pasterization; clarification of wine and beer; pretreatment stage for other membrane processes.	[11,12,17,28,29]
Ultrafiltration	ΔP^1^/Size exclusion	0.01–0.1	>5000	Low energy, easy operation and scale up, relatively low cost/Retention of only macromolecules and colloidal particles.	Protein fractionation andconcentrating; whey protein concentrating; recovery of valuable components.	[11,12,17,28,30]
Nanofiltration	ΔP^1^/Size, electric, and dielectric exclusion	0.001–0.01	500–2000	High efficiency, easy operation, lower energy and higher permeability compared with RO/High-cost operation and pressure, low retention of monovalent ions.	Lactose concentratingand demineralization; enzyme purification; fruit juice concentrating.	[5,12,28,31]
Reverse osmosis	ΔP^1^/Size, electric, and dielectric exclusion	<0.001	<500	High efficiency, easy operation, retention of all salts and monovalent ions, established process in large scale/High-cost operation and pressure.	Water desalination/demineralization; fruit juice concentrating; wastewater treatment.	[11,12,28,32,33,34,35]
Forward osmosis	ΔP^1^/Size, electric, and dielectric exclusion	<0.001	<500	Improved energy efficiency compared with RO/High-cost operation, more complex scaling formation compared with RO.	Water desalination/demineralization; fruit juice concentrating; wastewater treatment.	[12,33,35,36,37]
Electrodialysis	ΔE^2^ Electric exclusion	0.001–0.1	-	Higher rates of water recovery, reduced operational costs, simplified operation, and improved membrane stability compared with RO. Membrane degradation, not suitable for the separation of molecular compounds.	Concentrating saline solutions; wastewater treatment.	[11,12,28,34,38,39,40,41]
Membrane distillation	ΔP/ΔT^3^ Permeate condensation	0.01–1	-	Lower vapor space compared with traditional distillation columns, lower pressures and temperatures than the feed solution boiling point, high non-volatile solvent separating factor. Not commercialized yet, higher operational cost compared with RO.	Dehydration of aromatics and flavors; deacidification of fruit juices; concentrating dairy products; wastewater treatment.	[12,28,42,43]

**Table 2:** Processes involving modified membranes in the food and beverage industry: main improvements.

Filtration Processes	Liquid Studied	Membrane	Main Conclusions	References
Modified membrane	Pomegranate juice	Nitrogen plasma-reverse osmosis	Flow rate was three times higher than normal in clarification, saving time and obtaining 60°Brix.	[32]
	Green tea	Asymmetric alumina (Al_2_O_3_)–PES hollow fibers	The turbidity of green tea extract was reduced by 90% even after 30 days of refrigerated storage.	[133]
	Apple juice	PSF/PEI UF-TiO_2_and Al_2_O_3_nanoparticles	Improvement inantiscalant properties, with flow recovery above 90%, and in color, turbidity, total soluble solids, total phenolic content, and antioxidant capacity.	[134]
	Orange juice	GO layer to geopolymeric membrane	Exhibited the capability to concentrate the °Brix value by 1.73 times, showcasing promise in the field of fruit juice concentration	[135]
	Apple juice	Polysulfone UF–low-pressure oxygen plasma	Improved performance in clarification, enhancing hydrophilicity, and promoting better antiscaling behavior without the necessity for pretreatment steps.	[136]

## Data Availability

The original data presented in the study are openly available in this article.
